# Lessons Learned Recruiting and Retaining Pregnant and Postpartum Individuals in Digital Trials: Viewpoint

**DOI:** 10.2196/35320

**Published:** 2022-04-20

**Authors:** Amanda M Parks, Jennifer Duffecy, Jennifer E McCabe, Rachel Blankstein Breman, Jeannette Milgrom, Yafit Hirshler, Alan W Gemmill, Lisa S Segre, Jennifer N Felder, Lori Uscher-Pines

**Affiliations:** 1 Department of Psychology Virginia Commonwealth University Richmond, VA United States; 2 Department of Psychiatry College of Medicine University of Illinois at Chicago Chicago, IL United States; 3 Department of Psychology Western Washington University Bellingham, WA United States; 4 School of Nursing University of Maryland Baltimore, MD United States; 5 Parent-Infant Research Institute Austin Health University of Melbourne Melbourne Australia; 6 College of Nursing University of Iowa Iowa City, IA United States; 7 Osher Center for Integrative Health University of California San Francisco, CA United States; 8 Department of Psychiatry and Behavioral Sciences University of California San Francisco, CA United States; 9 RAND Corporation Arlington, VA United States

**Keywords:** digital trials, maternal and child health, pregnant and postpartum individuals, fraudulent enrollment, retention and recruitment, pediatrics, parenting, pregnant women, COVID-19, pandemic, postpartum, digital health

## Abstract

In an increasingly connected world and in the midst of a global pandemic, digital trials offer numerous advantages over traditional trials that rely on physical study sites. Digital trials have the potential to improve access to research and clinical treatments for the most vulnerable and minoritized, including pregnant and postpartum individuals. However, digital trials are underutilized in maternal and child health research, and there is limited evidence to inform the design and conduct of digital trials. Our research collaborative, consisting of 5 research teams in the U.S. and Australia, aimed to address this gap. We collaborated to share lessons learned from our experiences recruiting and retaining pregnant and postpartum individuals in digital trials of social and behavioral interventions. We first discuss the promise of digital trials in improving participation in research during the perinatal period, as well as the unique challenges they pose. Second, we present lessons learned from 12 completed and ongoing digital trials that have used platforms such as Ovia, Facebook, and Instagram for recruitment. Our trials evaluated interventions for breastfeeding, prenatal and postpartum depression, insomnia, decision making, and chronic pain. We focus on challenges and lessons learned in 3 key areas: (1) rapid recruitment of large samples with a diversity of minoritized identities, (2) retention of study participants in longitudinal studies, and (3) prevention of fraudulent enrollment. We offer concrete strategies that we pilot-tested to address these challenges. Strategies presented in this commentary can be incorporated, as well as formally evaluated, in future studies.

## Introduction

### Background

Although they have numerous benefits, digital trials are underutilized in maternal and child health research. Digital trials, sometimes referred to as internet, virtual, siteless, or decentralized trials, leverage technology to engage participants outside of physical sites, from recruitment through outcome assessment. Although digital trials first appeared in 2002, the COVID-19 pandemic and the need to protect research participants from exposure further spurred their growth. At present, digital trials are increasingly being used for clinical research, including drug trials and trials of social and behavioral interventions [1,2].

Although trials that are fully digital or incorporate digital elements can overcome some of the key limitations of traditional trials that rely on physical study sites, they also pose unique challenges for researchers and participants. Common concerns about digital trials include high attrition rates and the inability to reach research subjects with low digital literacy. Further, although methods have been developed for digital trials of drugs and devices [3], there is far less evidence to inform the design and conduct of digital trials of social and behavioral interventions among pregnant and postpartum individuals.

To address this evidence gap, a group of 5 research teams in the U.S. and Australia collaborated to share lessons learned conducting innovative digital trials with pregnant and postpartum individuals. Across our research collaborative, we recruited participants using multiple digital platforms, including platforms used by the general public (eg, Facebook, Instagram, Twitter) and those specifically targeting individuals in the perinatal period (eg, Ovia, BabyCenter). We conducted fully digital trials without any human interaction, as well as digital trials with some in-person or face-to-face synchronous interaction (eg, video visit to complete informed consent). In this commentary, we present lessons learned from 12 different completed and ongoing digital trials (Table 1) that evaluated interventions for breastfeeding, prenatal and postpartum depression, insomnia, decision making, and chronic pain. We focus on challenges and lessons learned in 3 key areas: (1) rapid recruitment of large samples with a diversity of minoritized identities, (2) retention of study participants in longitudinal studies, and (3) prevention of fraudulent enrollment. We offer concrete strategies in each of these areas through which we experimented. These strategies can be incorporated into as well as formally evaluated in future studies.

**Table 1:** Description of studies in the research collaborative.

Name of study, principal investigator(s)	Study status as of September 2021	Sample size, n	Completed studies: minoritized demographics, %	Ongoing studies: recruited and minoritized demographics as of January 1, 2022, n (%)	Study topic	Length of follow-up for longitudinal studies	Digital/social media platform used for recruitment	Retention, %
Prevention of Perinatal Depression, Dr Felder	Ongoing	Goal of 300	N/A^a^	Total: 280 Black: 27 (9.69); Asian/Pacific Islander: 26 (9.34); multiracial: 14 (4.84); other: 2 (0.69); Latinx/Hispanic: 35 (12.46)	Survey of pregnant adult women at risk for depression to evaluate the extent to which they are identified and referred for preventive intervention	N/A	Ovia	N/A
REST^b^ study, Dr Felder	Completed	208	Black, Asian/Pacific Islander, multiracial, other: 33.75; Latinx/Hispanic: 7.25; low income (<US $10,000/year): 32.2	N/A	Evaluation of digital CBT^c^ for insomnia among pregnant women	Enrolled up to 28 weeks gestation, followed until 6 months postpartum	Facebook, flyers, Research Match, word of mouth, University of California San Francisco (UCSF) electronic health record messages and patient letters	89
Beating the Blues before Birth, Drs Milgrom, Skouteris, Galbally, East, and Glover (Australia)	Ongoing	Goal of 230	N/A	Total: 63 Australia: 42 (66.67); New Zealand & Oceania: 4 (6.35); Europe: 7 (11.11); Africa: 2 (3.17); Asia: 2 (9.52); North America: 2 (3.17)	Evaluation of an antenatal depression treatment (CBT) on child neurodevelopment.	Mothers and children followed up to 24 months postbirth, additional follow-ups conducted at 10-week postrandomization, 3 months and 12 months postbirth	Ovia, Facebook, Parent Infant Research Institute Website	To be decided (TBD)
Sunnyside, Drs Duffecy and O’Hara	Completed	210	Black: 3.33; multiracial: 8.57; Asian: 2.86; Native Hawaiian/Pacific Islander: 0.95; Hispanic/Latinx: 11.90	N/A	Evaluation of digital intervention to prevent postpartum depression	Participants recruited at 20-28 weeks pregnant, remained in the trial until 12 weeks postpartum	Ovia, university email list (University of Illinois Chicago (UIC) and U of Iowa), Research Match	72
Sunnyside+, Drs Duffecy and Pezley	Completed	22	Black: 100; Latinx/Hispanic: 0.5; low income (<US $51,000/year): 54.5	N/A	Evaluation of digital intervention to prevent postpartum depression and improve breast feeding outcomes in Black women	Participants recruited at 20-28 weeks pregnant, remained in the trial until 12 weeks postpartum	Ovia	73
Sunnyside for prevention and treatment, Drs Duffecy and Maki	Ongoing	Goal of 90	N/A	Total: 48 Black: 3 (6.4); Asian: 2 (4.1); Native American: 2 (4.1); White: 36 (75); Latinx/Hispanic: 5 (10.4)	6-week digital intervention to prevent postpartum depression	6 weeks	Ovia, UIC clinic recruitment	TBD
Tele-MILC^d^, Dr Uscher-Pines	Ongoing	Goal of 2400	N/A	Total: 422 Black: 37 (8.77); Latinx/Hispanic: 72 (17.1); other minoritized race: 40 (9.48)	Evaluation of a breastfeeding support app	Participants recruited in their third trimester of pregnancy, remained in trial until infant 6 months old	Ovia	TBD
EPIC^e^ Survey, Dr McCabe	Ongoing	Goal of 150	N/A	Total: 106 Black: 13 (12.26); Native American: 2 (1.89); Asian/Pacific Islander: 9 (8.5); multiracial: 6 (5.66); Latinx/Hispanic: 12 (11.3)	Association between prenatal stressors during COVID-19 and subsequent child development	Wave 1 data at third trimester, wave 2 data at 6 weeks postpartum, wave 3 data at 16 weeks postpartum	Ovia, Facebook	TBD
Birth and Postpartum Care During COVID-19, Dr Breman	Completed	388	Asian/Pacific Islander: 3.2; Black: 7.1; mixed: 3.8; Latinx/Hispanic: 11.6; low income (<US $50,000/year): 16	N/A	Experience of giving birth and postpartum care during the first wave of the COVID-19 pandemic	N/A	Ovia	N/A
Shared Decision-Making During Hospital Birth, Dr Breman	Completed	1173	Asian/Pacific Islander: 6; Black: 10.4; mixed: 5.4; Latinx/Hispanic: 13; low income (<US $50,000/year): 26	N/A	Assessment of shared decision making during hospital birth in the U.S.	N/A	Ovia, Pacify, Facebook/Instagram, YouTube	N/A
BetterLife, Drs Vignato and Segre	Completed	158	Black: 10.76; Native American: 1.27; Latinx/Hispanic: 13.29; Asian/Pacific Islander: 1.90; other/2 or more races: 3.80; low income (Medicaid eligible): 32.28		Assessment of the relationship between low back and pelvic pain, depression symptoms, and quality of life in pregnant women in their third trimester	N/A	Ovia	N/A
Listening Visits for NICU^f^ mothers, Dr Segre	Ongoing	29	N/A	Total: 4; no racially or ethnically minoritized participants	Evaluation of listening visits delivered via Zoom by NICU nurses to emotionally distressed mothers of hospitalized newborns	Participants recruited while their newborns are hospitalized	Ovia	TBD

^a^N/A: not applicable.

^b^REST: Research on Expecting Moms and Sleep Therapy.

^c^CBT: cognitive behavioral therapy.

^d^Tele-MILC: Telehealth to Increase Mothers’ Lactation Confidence.

^e^EPIC: Experiences of Pregnancy and Isolation during COVID-19.

^f^NICU: neonatal intensive care unit.

### The Promise of Digital Trials for Pregnant and Postpartum Individuals

Digital trials have numerous benefits**.** First, they offer an alternative to costly and inconvenient traditional trials [4,5]. Traditional trials, with their multiple limitations, have dominated the landscape since the 1940s but needed disruptive innovation [6]. Participants in traditional trials must be located near physical study sites, restricting access for many. Further, it is well documented that most traditional trials fail to meet their recruitment targets [7,8]. Traditional trials are also expensive [4,5].

In the area of access and participation, digital trials can support rapid recruitment of large samples [1]. Because participants do not need to be near a study site, these trials can serve hard-to-reach and diverse populations [1,9]. Further, because digital trials offer participants greater autonomy, convenience, and privacy, they may be more appealing to certain participants who would not otherwise engage [10]. Researchers at Harvard and the Massachusetts Institute of Technology (MIT) demonstrated that digital trials may improve access to studies for women and racially and ethnically minoritized populations, who are significantly underrepresented in clinical trials [11,12].

With regard to costs, digital trials are also likely to be more efficient because they require smaller teams of investigators [2] and avoid power reduction due to clustering, which is an issue when recruiting from multiple sites [13]. Further, digital technologies, which allow for continuous data collection or data collection at more time points, can reduce costs related to clinical assessments [14].

Furthermore, digital trials introduce a host of methodological advantages. For example, with electronic consent (eConsent) procedures, multimedia web tools (eg, videos, animation) can be used to enhance understanding [15], and randomization has the potential to be more secure [2,16].

Although many populations can benefit from digital trials, they may be particularly suited for pregnant and postpartum individuals. First, demands of infant care can make travel challenging, and studies have demonstrated that young parents find it difficult to visit clinical sites to participate in research [17]. Second, the perinatal period is one that is rife with distress, with approximately 20% of childbearing women exhibiting symptoms of anxiety and depression [18,19]. The significant responsibilities and physical and emotional changes that occur in the perinatal period often impede individuals from engaging in healthy behaviors and participating in research that may benefit themselves or science in general [17]. Third, women of childbearing age exhibit the highest rates of smartphone ownership [20]. As such, the historic criticism that requiring internet use may lead to less representative samples in digital trials may be not be applicable to this population [2,16,21].

Although many of these advantages were clear prior to the COVID-19 pandemic, the pandemic revealed additional benefits with great urgency and led to the rapid adoption of digital engagement strategies. The social distancing orders in March 2020 led the US Food and Drug Administration to issue guidance on the safety risks of proceeding with traditional trials and urged researchers to develop safer alternatives for data collection [10]. Shortly thereafter, a review by ClinicalTrials.gov revealed that patient interactions in ongoing trials, including some focused on pregnant and postpartum populations, began to predominantly occur remotely [22]. This shift to digital engagement is expected to persist. Most clinical trial investigators expect a threefold increase in digital patient interactions 6 months postpandemic [22,23].

### Digital Trials and Tribulations

Digital trials of social and behavioral interventions, while innovative, also face unique challenges. First, a key concern is that because these studies leverage technology, they cannot engage individuals without mobile devices or access to the internet. Further, participants must have digital literacy (eg, to complete online assessments, download a study app). These requirements may lead to a lack of representation and may perpetuate health disparities, as minoritized and underserved populations have reduced broadband access and subsequently less health and digital literacy [23]. For example, as of April 2021, 80% of White Americans had home internet access compared to 71% of Black and 65% of Latinx Americans [20]. Second, attrition in longitudinal studies remains a serious concern. Research has shown that digital trials have higher attrition rates, in part, because research subjects are not as invested or activated. In addition, the personal, human touch that occurs during in-person interactions with members of the study team, lacking with digital trials, may be a key ingredient for retention. Lastly, although privacy has been noted to be a strength of digital trials, it can also be a limitation. Although digital trials allow a certain level of anonymity, trials that occur in a participant’s home over the internet may face challenges with data security. In sum, securing participant data and ensuring privacy are challenges, and researchers must continue to develop methods to monitor and evaluate data from health technologies [24].

## Key Challenges and Useful Strategies

### Background

In the past 5 years, our research teams have launched numerous digital trials as well as modified existing traditional trials among pregnant and postpartum individuals to incorporate digital trial elements. We have used several social media platforms and pregnancy and parenting apps for recruitment, with the most common being Facebook and Ovia. Facebook is the most popular social media platform among American adults, with 69% reporting that they use Facebook [20]. Facebook is popular across all demographic groups; however, some adults access it more often. Specifically, 77% of women use Facebook daily compared to 61% of men, and 74% of Black Americans use Facebook daily compared to 67% of White and 72% of Latinx users. Ovia is 1 of the most popular pregnancy apps available for free download [25]. Used by over 2 million people in the U.S. each month (email communication with Ovia staff, August 10, 2021), it provides educational content, conducts health assessments, and uses proprietary algorithms and machine learning to provide user-specific support, advice, and resources [25].

Although we confronted numerous challenges in designing and executing our digital trials, we found 3 areas to be particularly daunting: rapid recruitment of large, diverse samples; retention; and fraudulent enrollment monitoring. We explain each of these areas next, as well as promising strategies to overcome the challenges.

### Challenge #1: Rapid Recruitment of Large, Diverse Samples

Across all the social media platforms and apps, our studies were typically featured in a paid ad. Potentially interested participants saw the ad, clicked on it, and were routed to a study web page or screening survey. Although the steps varied by study, many participants then completed an eligibility screening survey, completed an informed consent process, and were enrolled. In this recruitment process, we struggled with recruiting minorized individuals and routing eligible participants through the enrollment process.

#### Minoritized Participants

None of the platforms we used for recruitment allowed us to target ads to users of a particular race or ethnicity. However, it was possible to target based on geography (eg, state or zip code), and in the case of Ovia, the stage of pregnancy and parity. In August 2020, after several lawsuits and scandals surrounding housing discrimination by advertisers, Facebook no longer permitted targeting based on race [26,27]. Given this new policy, researchers in our collaborative were unable to use ads to directly oversample minoritized groups. To overcome this constraint, the Research on Expecting Moms and Sleep Therapy (REST) study, a longitudinal study that examined the effectiveness of digital cognitive behavioral therapy (CBT) for insomnia for pregnant women, used the Facebook audience tool to advertise to certain zip codes that have a high proportion of Black and Latinx populations.

Further, even in cases where a platform’s user base is nationally representative and ads go out to users of all races and ethnicities, we learned that we may fail to generate interest among minoritized groups. For example, in several studies, we found that highly educated, White participants were more likely to click on study ads and were disproportionately represented. As a result, our teams worried that we may be perpetuating the systemic barriers that minoritized and marginalized groups face in accessing clinical research and care. To combat this, we experimented with the following strategies:

- Strategy 1: Expand beyond paid ads. Although researchers can pay platforms to advertise, there are other ways to reach minoritized populations on social media. Members of our collaborative used social media platforms to join online pregnancy support groups (eg, Black Mamas Matter, Black Families Do Breastfeed) and promote the study if given approval. For example, members of the CHOICE for Birth study team used professional and personal networks to contact and collaborate with an influencer to promote their study. Further, the team used a recruitment firm to connect them with accounts on social media that were specifically tailored to minoritized groups. Through this connection, the team was able to have a paid ad on Instagram and successfully recruited more Asian pregnant people.
- Strategy 2: Run ads that feature images of racially or ethnically minoritized pregnant people and explicitly state in the materials that the research team is recruiting minorized populations. This strategy falls under the category of surface structure adaptations (vs deep adaptations, which demonstrate the salience of the intervention for the target population) [28]. Here, visual modifications to the materials and intervention content are implemented based on more superficial characteristics (eg, locations, language, food) of or preferred by the target group. These strategies demonstrate how the research program or materials fit with the culture and may increase acceptance of the materials [28]. Most researchers in our collaborative utilized these types of strategies. The Telehealth to Increase Mothers’ Lactation Confidence (Tele-MILC) study, a National Institutes of Health (NIH)-funded trial to assess the impact of a breastfeeding app, utilizes this strategy, among others. Although the study continues to recruit, as of September 2021, two-fifths (n=80, 40%) of the 200 enrolled participants identified as Latinx or Black.
- Strategy 3: Partner with community members to develop culturally concordant recruitment materials. For example, 1 of our research teams is planning to partner with a participant recruitment program that has services to support enrollment of underrepresented populations. The program will collaborate with community members, who will provide feedback on recruitment materials. They will then offer consulting services to ensure materials are in plain language.

#### Sustaining Interest Through the Informed Consent Process

Some of our digital trials lost large numbers of eligible individuals during the recruitment process because of the time-consuming, intimidating, and non-user-friendly nature of the informed consent process. For example, in a trial focused on parents of hospitalized newborns that used Ovia for recruitment, 40% (n=72) of individuals who viewed the study ad were eligible to complete the eligibility screening survey, and 33% (n=24) completed the eligibility screening survey. However, only two individuals (18% of those who were deemed eligible to enroll in open trial) completed the eConsent form over the entire recruitment period for Ovia. The research team attributed this low enrollment to the use of a long and legalistic informed consent form typically used for face-to-face recruitment. Although our teams seldom received direct, formal feedback from participants initially, several teams that changed their consent processes to be more streamlined saw immediate results.

Our teams implemented several strategies to improve the eConsent experience for participants. First, 1 team had initially included a 2-part consent process, where potential participants had to consent to the screening survey and then, if eligible, the full study. In the streamlined version, the team is planning to have a single consent task that occurs following screening.

Another team with challenges enrolling participants revised the look and content of the eConsent form. Members of the Encouraging Mothers and Babies Everywhere - Research Center (EMBERcenter) [29] revised their eConsent process after consulting with a marketing strategist. They inferred that the original consent process was problematic, given the limited number of eligible participants who successfully completed the full consent process. Progress of potential participants was tracked using Qualtrics. After consultation with the marketing strategist, the EMBERcenter team modified the informed consent document to be a letter from the principal investigator and included emojis, a picture of the principal investigator, and bullet points in place of some paragraphs. They also cut some material so that the final version read more like a description a participant might hear from a research coordinator enrolling participants in person.

The goal of many of these strategies is to provide a seamless experience for the participant as they leave the social media or app platform and enter the virtual study environment. If the social media platform is informal and has limited text, then the study environment should mirror that as much as possible. We recommend that researchers conducting lower-risk digital trials work with their institutional review board (IRB) to amend the consent length and requirements. The goal is to utilize as few words as possible to ensure that participants understand the risks and benefits and to avoid creating consent materials that look like a technology company’s terms of service, given many users are accustomed to signing these without reading them. Because many guidelines and templated consent documents are based on traditional in-person trials, research teams may need to work with and educate their IRBs about adaptations for digital trials.

### Challenge #2: Retention

Once participants are successfully enrolled, it can be difficult to retain them. In many of our studies, we required participants to respond to online surveys over months or years, and we were concerned that high attrition rates would pose a threat to validity. As previously stated, attrition is a well-documented problem in digital trials, with most participants dropping out within the first week of the study [30]. Fortunately, robust engagement strategies can have a significant impact on retention. Several digital trials across our study teams had above-average retention rates [31,32].

Our study teams implemented several novel retention strategies beyond providing incentives and sending reminders to reduce attrition. One effective strategy for traditional trials is a 30-minute orientation to the study prior to randomization [33]. During the interactive orientation, motivational interviewing techniques can be used to process feelings and ambivalence about the intervention and the different groups participants could be assigned (ie, control vs experimental) [33]. In the REST study [34], these orientations were conducted by a study coordinator via phone. In the Sunnyside study, an evaluation of a longitudinal digital intervention using CBT to prevent the development of postpartum depression, an initial engagement call was completed to introduce participants to the program and ensure ability to access study materials [35].

Another strategy 1 of our teams used was to include escalating incentives based on the proportion of online surveys that the participants completed. Further, some study teams sent gifts to participants to demonstrate the importance and value of the participants’ information and participation. For example, in the Experiences of Pregnancy and Isolation during COVID-19 (EPIC) Survey, the research team mailed baby wipes along with a letter to congratulate participants on the birth of their baby. The Tele-MILC study engaged participants by having monthly contests that were announced in the study’s newsletter; specifically, participants could create memes, send in a baby photo, or answer a trivia question to receive additional incentives.

Other strategies to improve engagement focused on creating a community among participants and eliciting altruism. The study team for the EPIC Survey implemented a few strategies to create a community feeling. First, they gave a group name to their participants so that a more personal feeling was elicited. Further, study staff generated a map of all zip codes where participants lived in order to show the reach of the study and proximity to other participants. The team placed a dot near each zip code where a participant lived in order to ensure privacy and confidentiality. Members of the Tele-MILC study team made a “Thank You” video, which appeared at the end of the enrollment process. In the video, the principal investigator and members of the research team conveyed their appreciation of the participants, the importance of the study, and the role that participants were playing in contributing to science. The video was designed both to tap into the participants’ altruism and to show the real humans behind the digital trial.

### Challenge #3: Fraudulent Enrollment

Fraudulent enrollment is a common problem in digital trials, particularly trials that utilize social media for recruitment, and can introduce threats to data integrity and sample validity [36]. Fraudulent enrollment can occur in several ways. First, ineligible individuals can misrepresent themselves as eligible. In some of our studies, we were concerned that men or women who were not pregnant at the time of recruitment would attempt to enroll despite ads clearly describing the target population. We were also concerned that certain individuals would continue to edit their responses to the screening survey until the instrument declared that they were eligible. Second, participants who are eligible can attempt to enroll in a study more than once to obtain additional incentives. Lastly, software applications that run automated tasks, known as bots, can pose as participants to receive incentives [37]. Several of our study teams have monitored for fraudulent enrollment and detected bots. We found that an advantage of Ovia, and other platforms that target pregnant and postpartum people, is that there are fewer instances of ineligible individuals trying to enroll.

Research teams that used social media platforms that were not used exclusively by pregnant and postpartum individuals developed methods to verify pregnancy and identify bots. Some teams requested photos of a recent ultrasound or required that the participants upload a birth certificate. One team included an “insider” question (ie, a question that only an eligible participant is likely to answer correctly) in an early survey, asking each participant to enter the name of their obstetrician.

Additional strategies that our digital trials used to detect fraud included consistency checks (eg, Does “weeks pregnant” match the baby’s due date?) and open-ended survey questions. Open-ended questions are useful because the study team can assess whether answers are coherent. The Tele-MILC study combined 2 fraud detection strategies (insider and open ended) in 1 survey question. In the first survey, we asked individuals what they liked most about the Ovia app. We reviewed responses to this question to ensure that participants were in fact Ovia users (and that off-platform recruitment was not taking place) and were actual humans versus bots with incoherent answers. Additional strategies are included in Table 2.

**Table 2:** Concrete strategies for improving recruitment, retention, and fraud monitoring.

Strategy		Description
**Recruitment**		
	Expand beyond paid ads.	Utilize social media influencers to promote your study. Join groups that are dedicated to your population (eg, Black Mamas Matter) and ask permission to promote the study.
	Run targeted ads.	Create ads that feature images of racially or ethnically minoritized individuals or your targeted population of interest to improve acceptability and signal that your study is interested in their experiences.
	Develop culturally concordant materials.	Utilize university or external programs that have services to support enrollment of underrepresented populations that partner with community members to create or contribute to materials. Employ and collaborate with community members. Before enrollment or conceptualization of your study, engage with your community of interest and develop a relationship. Understand their needs and wants in advance and reflect on your positionality and privilege as a researcher before undertaking the research study.
	Improve the eConsent^a^ process.	Condense the consent process into 1 step. Edit the consent form to include more visual elements and highlighted bulleted points. Streamline the process from the ad to the study landing page. Ensure that your website matches the style and language of the social media platform as much as possible. Meet with your IRB^b^ to amend the requirements on length and detail.
	Utilize experts from other disciplines (eg, marketing).	Hire marketing strategists or other communications experts to review and edit your study materials, particularly consent and assent documents, to ensure they are digestible and inviting
**Retention**		
	Collect secondary contacts.	Collect a friend or relative’s contact information in case the research team loses touch with the participant.
	Send birthday congratulations.	Email or mail birthday cards and other postcards for important milestones.
	Send monthly newsletters.	Send participants a monthly newsletter that contains descriptions or interviews with a member of the research team and other helpful resources related to the study topic (eg, ideas for self-care, fun facts about pregnancy).
	Share positive quotes.	Ask participants to share quotes about their experiences in the study to share widely with other participants.
	Send reminders.	Utilize an automated service to send reminders to participants about their upcoming assessments or have a research team member text, call, or email personalized reminders.
	Provide escalating incentives.	Provide different levels of incentives/compensation based on how many follow-up surveys the participant completes. Make the final assessment worth more than prior assessments.
	Utilize games/contests.	Create contests wherein participants can be compensated or entered into a raffle to win a gift card or other incentive. Contests such as “best caption for a meme or gif” and solving riddles have been engaging.
	Send gifts.	Send participants items (eg, coffee mugs, pens, notebooks, baby wipe case, hand sanitizer) with the study name or logo on it.
	Develop a video from the study team.	Create a thank-you video to appear at the end of a survey or on your study website, featuring the research team and positive messages or stories to elicit altruism.
	Create opportunities for networking/interaction.	Create a visual map of participants’ locations. Create a social media page where study participants can interact; host virtual meetings for participants to facilitate interactions among them.
**Fraudulent enrolment**		
	Check internet protocol (IP) addresses or latitude and longitude.	Review IP addresses (unique addresses that identify devices on the internet or on a local network) to ensure that the same individual is not attempting to enroll more than once.
	Add reCAPTCHA (Google).	Add reCAPTCHA, a tool that uses advanced risk analysis techniques, to distinguish between humans and bots.
	Require face-to-face meetings or emailing back and forth.	Require that potential participants engage with the study team (either in a synchronous meeting or via asynchronous communication) prior to dispensing incentives.
	Only dispense incentives after eligibility is confirmed.	Do not automatically dispense incentives; ensure fraudulent enrollment monitoring is completed before participants are official enrolled in order to keep bots and ineligible participants from depleting your incentives.
	Include honeypot questions.	Include a survey question that is invisible to humans but visible to bots. If it is answered, this suggests bot activity.
	Perform consistency checks.	Include 2 or more survey questions that ask the same question in different ways (eg, age and date of birth); check for consistent responses.
	Include insider questions.	Include a question that only an eligible participant is likely to answer correctly/know the answer. A common example is to ask members of the military a question about their rank.
	Include time stamps/time to complete survey.	Include time stamps and review how long it takes for a participant to complete a survey. For example, flag if the participant completes a long survey in less than 5 minutes.
	Create a duplicate email flag.	Flag if the same email is entered in the enrollment records of multiple participants.
	Control survey navigation.	Do not enable a back button in screening surveys. Including a back button may enable participants to change prior answers to meet eligibility criteria.

^a^eConsent: electronic consent.

^b^IRB: institutional review board.

## Conclusions

Despite the demonstrated need and utility of digital trials for pregnant and postpartum individuals, the guidance on methodology remains limited. Methods are needed for the recruitment and retention of large, diverse samples, particularly minoritized populations, given the systemic barriers these communities face in participating in research. As such, our collaborative aimed to begin a dialogue and generate recommendations for researchers as well as reviewers of digital trial protocols. Although the strategies we presented in this commentary have been pilot-tested in 1 or more trials, future research should formally test their effectiveness with different populations and study types. Given the rapid growth and important advantages of digital trials, strong study designs that help to overcome their weaknesses are needed to advance the science and spur ongoing innovation in the field of maternal and child health.

## Abbreviations

CBT: cognitive behavioral therapy
eConsent: electronic consent
EMBERcenter: Encouraging Mothers and Babies Everywhere - Research Center
EPIC: Experiences of Pregnancy and Isolation during COVID-19
IRB: institutional review board
REST: Research on Expecting Moms and Sleep Therapy
Tele-MILC: Telehealth to Increase Mothers’ Lactation Confidence
